# Using Antimicrobial Photodynamic Therapy with Ultrasound Devices and Bioactive Glasses as a Combined Approach for Treating Dentin Caries Lesions

**DOI:** 10.3390/pathogens12081052

**Published:** 2023-08-17

**Authors:** João Felipe Besegato, Priscila Borges Gobbo de Melo, Adilson César Abreu Bernardi, Marina Trevelin Souza, Edgar Dutra Zanotto, Vanderlei Salvador Bagnato, Alessandra Nara de Souza Rastelli

**Affiliations:** 1 Department of Restorative Dentistry, School of Dentistry, Araraquara, São Paulo State University—UNESP, Araraquara 14801-903, SP, Brazil; jfbesegato@gmail.com (J.F.B.); pribgobbo@gmail.com (P.B.G.d.M.); 2 Department of Biology and Health Sciences, University of Araraquara—UNIARA, Araraquara 14801-340, SP, Brazil; acabernardi@gmail.com; 3 Vitreous Materials Laboratory (LaMaV), Department of Materials Engineering, Federal University of São Carlos—UFSCar, São Carlos 13565-905, SP, Brazil; marina.trevelin@gmail.com (M.T.S.); dedz@ufscar.br (E.D.Z.); 4 Department of Materials Science and Physics, Physics Institute of São Carlos, University of São Paulo—USP, São Carlos 13566-590, SP, Brazil; vander@ifsc.usp.br

**Keywords:** dental caries, biofilm, caries removal

## Abstract

Novel approaches for caries lesion removal and treatment have been proposed. This study evaluates the combined use of an experimental ultrasound, aPDT (antimicrobial photodynamic therapy) and bioactive glasses on the removal, decontamination and remineralization of dentin caries lesions. A biological model created with a duo species biofilm (*Streptococcus mutans* and *Lactobacillus acidophilus*) was used for the development of a caries-like lesion over the dentin for 7 days. Bovine dentin specimens (4 × 4 × 2 mm) were randomized according to the following caries removal techniques: bur (BUR) or ultrasound (ULT), decontamination (with or without aPDT) and remineralization materials (45S5 or F18 bioactive glasses). The following different groups were investigated: caries lesion (control); sound dentin (control); BUR; BUR + aPDT; ULT; ULT + aPDT; BUR + 45S5, BUR + F18; ULT + 45S5; ULT + F18; BUR + aPDT + 45S5; BUR + aPDT + F18; ULT + aPDT + 45S5; and ULT + aPDT + F18. Transverse microradiography (TMR), cross-sectional microhardness (CSH), FT-Raman spectroscopy and confocal microscopy (CLSM) were performed. A two-way ANOVA and Tukey’s test were used (α = 0.05). (3) Results: The TMR revealed a lesion depth of 213.9 ± 49.5 μm and a mineral loss of 4929.3% vol.μm. The CSH increases as a function of depth, regardless of the group (*p* < 0.05). Removal with BUR (24.40–63.03 KHN) has a greater CSH than ULT (20.01–47.53 KHN; *p* < 0.05). aPDT did not affect the CSH (*p* > 0.05). No difference was observed between 45S5 or F18 (*p* > 0.05), but a change was observed for ULT (*p* > 0.05). The FT-Raman shows no differences for the phosphate (*p* > 0.05), but a difference is observed for the carbonate and C-H bonds. The CLSM images show that aPDT effectively inactivates residual bacteria. A combination of ULT, aPDT and bioactive glasses can be a promising minimally invasive treatment.

## 1. Introduction

Dental caries is a biofilm-mediated, diet-modulated, multifactorial and non-communicable dynamic disease [1,2] influenced by behavioral, psychosocial and environmental factors [2], affecting all age groups globally [3].

Currently, a minimally invasive treatment of caries lesions employs selective caries-affected dentin removal. The purpose of this minimally invasive treatment is to remove only the caries-infected dentin, and the affected dentin, which is capable of remineralization, is preserved [4,5]. However, the clinical differentiation of these two layers (infected and affected dentin) is quite subjective, mainly in deep lesions, which can result in the unnecessary removal of the caries-affected dentin [6]. Once caries-like dentin removal is required, usually, round carbide burs under a low-speed rotation are widely used. However, these rotatory instruments tend to promote excessive and uncontrolled removal [7]. Based on these limitations, ultrasound-driven devices can be used as a minimally invasive approach [8,9,10,11], since the oscillatory vibration may promote conservative cavity preparation [9] and, consequently, less excessive removal of the caries-affected dentin.

Besides the minimal removal of the caries-affected dentin, the eradication of or reduction in the cariogenic pathogens load is important once the preserved affected dentin contains a bacterial load, which may lead to recurrent infection including pulp sensitivity, recurrent caries and pulpal inflammation [6,12,13,14]. Different antibacterial agents, such as triclosan, cetylpyridinium chloride, povidone iodine, hydrogen peroxide, sodium hypochlorite and chlorhexidine gluconate (CHX) have been used alone or in different dental products [13,15,16,17,18]. Chlorhexidine is the most commonly used cavity disinfectant [13]. Despite its beneficial effects, other antimicrobial agents and techniques have been proposed as cavity disinfectants [15,16,17,18]. In this way, antimicrobial photodynamic therapy (aPDT) has been used as a promising antibacterial technique against cariogenic pathogens, mainly in the management of deep lesions [6,18,19,20,21]. The aPDT reaction is based on the interaction among three components: a non-toxic molecule (photosensitizer), a light source under a wavelength that is capable of exciting the photosensitizer, and local oxygen molecules [22,23]. The absorption of photons by the photosensitizer triggers the formation of free radicals and reactive oxygen species (ROS), especially singlet oxygen [24]. These highly reactive and toxic oxygen species are able to damage proteins, lipids, nucleic acid and other cellular structures, resulting in cell damage and death [25]. Thus, it is assumed that aPDT unlikely promotes antibacterial resistance since oxygen species non-selectively interact with the bacteria structures via different metabolic pathways [26,27,28]. Moreover, aPDT is a highly selective technique since only cells containing photosensitizers and cells that are irradiated by the light are affected [29].

Besides the conservative caries-like dentin removal where the affected dentin is preserved and decontaminated, the use of biomaterials to remineralize the remaining dentin has been investigated [30,31]. Bioactive materials, such as bioactive glasses, have been used as remineralizing and desensitizing agents [32,33]. Although their use has primarily focused on bone regeneration, the similarity of composition between the bone and the other dental tissues suggests that bioactive glasses can be effective in regenerating enamel and dentin [32]. The remineralization potential is attributed to the ion release from the bioactive glass matrix followed by the precipitation and formation of hydroxyapatite [34]. However, the remineralization efficacy and the required time for that to occur are still uncertain.

Based on the advantages of ultrasound devices, aPDT and bioactive glasses, the combined use of these techniques/materials may result in a minimally invasive approach for caries lesion treatment. Although, individually, these techniques are already used in dentistry, to the best of our knowledge, the effect of the combination of these techniques has not been investigated. Thus, the purpose of this study is to evaluate the mechanical, chemical and antibacterial effects of combining ultrasound devices, curcumin-mediated aPDT and bioactive glass applications to remove, decontaminate and remineralize caries-like dentins, respectively. The null hypotheses tested were that the different approaches provided: (i) no differences in the dentin’s cross-sectional hardness; (ii) no differences in the organic and mineral content of the dentin and (iii) no differences in the bacterial viability over the residual dentin.

## 2. Materials and Methods

### 2.1. Experimental Design

This is an in vitro study with independent variables including the methods of caries removal (carbide bur or ultrasound device), cavity decontamination (with or without aPDT application) and dentin remineralization (application of 45S5 or F-18 bioactive glasses). The dependent variables were the cross-sectional microhardness of the dentin, the organic and mineral content of the dentin and the bacterial viability over the residual dentin after treatments. Table 1 shows the investigated groups and interventions.

### 2.2. Specimen Preparation

Bovine incisor teeth (n = 183) were used under proper ethical approval by the Ethics Committee for Animal Use from the School of Dentistry, Araraquara, Sao Paulo State University—UNESP (protocol number: 27/2018). The teeth were properly cleaned with pumice stone powder mixed with water to make a paste, and stored under 0.1% (*w/v*) thymol solution until the beginning of the experiments. All teeth were analyzed using a stereomicroscope (SZ61, Olympus Corporation, Tokyo, Japan) loupe under 10× magnification in order to select only those without visible structure defects and/or cracks on the enamel.

After selection, the teeth were sectioned 1 mm above the amelocemental junction to separate the crown from the root using a diamond wafering blade (11-4244, 0.3 mm, 15 HC, Buehler Wafering Blade, Buehler Ltd., Lake Bluff, IL, USA) coupled in a water-cooled precision cutting machine (IsoMet 1000, Buehler Ltd., Lake Bluff, IL, USA) under 250 rpm. Subsequently, enamel/dentin specimens of 4 × 4 mm were obtained, and the dental enamel was removed using silicon carbide abrasive papers (#80-grit, Wetordry™, 3M™, St. Paul, MN, USA) coupled in a politrix to obtain only bovine dentin specimens of 4 × 4 × 2 mm. After that, the dentin specimens were polished with silicon carbide papers via decreasing granulation (#600 #1200 and #1500-grid, Wetordry™, 3M™, St. Paul, MN, USA).

To standardize and avoid specimen bias, the surface Knoop microhardness was measured before the experiments. For this, three indentations were made at the center of the dentin surface using a Knoop microhardness testing machine (HMV-2, Shimadzu, Japan) with 10 g for 10 s [35]. Only the specimens with 40.86 ± 8.74 KHN were included in the study. The specimens were stored in distilled water under refrigeration at 8 °C and refreshed every 7 days until the beginning of the experiments.

### 2.3. Development of Caries-like Dentin

The dentin specimens were individually and vertically suspended in a 24-well culture plate using a modified active attachment model. The specimens were fixed over circular glass coverslips (K5-0013, KASVI, São José dos Pinhais, PR, Brazil) using a light viscosity condensation silicon (Oranwash L, Zhermack SpA, Badia Polesine, RO, Italy) [36]. After that, the glass coverslips containing the specimens were fixed on an orthodontic wire to keep each specimen suspended in the well to prevent the gravity action on bacterial colonization [36]. The whole set (glass coverslips, dentin specimens and orthodontic wire) was immediately autoclaved at 121 °C for 15 min and then transferred to a 24-well culture plate [36].

For the caries-like dentin development, the specimens were immersed in a bacterial suspension containing 1 mL of brain heart infusion (KASVI, São José dos Pinhais, PR, Brazil) and 1 mL of Lactobacilli MRS broth (Becton, Dickinson and Company, Franklin Lakes, NJ, USA); culture media were both supplemented with yeast extract 0.5 g/100 mL, glucose 1 g/100 mL and sucrose PA (pro-analysis) 2 g/100 mL. For each 50 mL of caries solution, 5 mL of *Lactobacillus acidophilus* (ATCC #ITAL-523) and 5 mL of *Streptococcus mutans* (ATCC #25175) at 10^8^ CFU/mL were added to induce bacterial colonization [19]. The 24-well plates were maintained under microaerophilic atmosphere at 37 °C for 7 days [37], with the culture media refreshed every 24 h. The pH of the caries solution was measured after the culture media were refreshed (pH 4.0) using pH indicator strips (pH-Fix 0-14, Macherey-Nagel GmbH & Co. KG, Düren, Germany).

### 2.4. Caries Lesion Removal

After the caries-like dentin induction, the specimens were rinsed with distilled water, and a dye solution (Revelacarie, ASFER, Indústria Quimica Ltda, Sao Caetano do Sul, SP, Brazil; batch number: 2450) was applied over the dentin for 10 s to identify the infected and affected dentins according to the criteria described by Momoi et al. [38]. The specimens were fixed and stabilized in a glass plate using a utility wax. Exploratory probe, dentin hardness and visual analysis were used and considered during the caries removal. Artificial spotlights were used to illuminate the operatory field, being considered adequate by the operator to perform the procedures. The experiments were performed in aseptic conditions. All the procedures were performed by a single operator to avoid inter-operator variability.

Carbide bur: The caries-like dentin lesion removal was performed using a carbide bur (AR 7, JET Carbide Burs, American Burrs, Palhoça, SC, Brazil) coupled with a low-speed handpiece (LB100, Beltec Ind. E Com. de Equipamentos Odontologicos, Araraquara, SP, Brazil; serial number: LB1-0132680). The parameters used were 15,000 rpm, power of 40 W and frequency of 0.06 kHz.

Ultrasound device: The caries-like dentin lesion removal was performed using a spherical diamond-coated tip (E3D, Helse Dental Technology, Santa Rosa de Viterbo, SP, Brazil; batch number: 17080618) coupled with an ultrasound device developed by the Physics Institute of Sao Carlos from the University of Sao Paulo—USP under constant distilled water cooling. A power of 30 W and a frequency of 27.5 kHz were used.

### 2.5. Cavity Decontamination Using aPDT

For the groups that received aPDT application as a cavity decontamination method, curcumin was synthetized according to the method described by Carmona-Vargas et al. [39] and used as a photosensitizer. The work solution of curcumin (270 µM) was solubilized in dimethyl sulfoxide (1% DMSO; Merck KGaA, Darmstadt, Germany; batch number: SHBF7360V). After that, the specimens were individually immersed in 1 mL of curcumin solution and kept in the dark for 5 min (incubation/pre-irradiation time). After that, the specimens were uniformly and simultaneously irradiated with a blue LED (BioTable, MMOptics, São Carlos, SP, Brazil) at a wavelength of 460 nm under a light dose of 15 J/cm^2^, with an irradiance of 22 mW/cm^2^ and irradiation time of 11 min and 36 s [40].

### 2.6. Dentin Remineralization Using Bioactive Glasses

To promote remineralization of the residual dentin (affected dentin) after caries-like dentin removal, two types of bioactive glasses were used, Bioglass^®^ 45S5 (SiO_2_-Na_2_O-CaO-P_2_O_5_) and F18 (SiO_2_-Na_2_O-K_2_CO_3_-CaO-MgO-P_2_O_5_), both provided by Vetra Ltda. (Ribeirão Preto, SP, Brazil) in collaboration with the Vitreous Material Laboratory from the Department of Material Engineering, Federal University of Sao Carlos—UFSCar, Sao Carlos, SP, Brazil). The bioactive glasses were ground in an agate jar using a Pulverisette mill (FRITSCH GmbH—Milling and Sizing, Idar-Oberstein, Germany) and sieved in a nylon sieve to obtain particles smaller than 45 μm (D_50_ = 5 microns). Both materials were incorporated into a cellulose gel at a concentration of 25% (0.175 mg of bioglass powder into 0.500 mg of cellulose gel) to facilitate the handling and application over the dentin surface. The bioactive glasses were applied over the dentin for 5 min [41,42] using a disposable microbrush.

### 2.7. Transversal Microradiography (TMR)

The mineral loss and caries-like lesion depth were evaluated via TMR images. The dentin specimens (n = 15) were longitudinally and perpendicularly sectioned to obtain dentin slices no more than 100 µm, which is the maximum allowed thickness for TMR analysis. To verify the slice thickness, a digital micrometer (MDC Lite 293-821-30, Mitutoyo Sul Americana Ltda., Suzano, SP, Brazil) was used. Previously, to obtain the microradiographs, the slices were kept in a humid environment to avoid dehydration and structural damage during X-ray exposure [43]. The slices were placed on a sample holder in combination with a step-wedge calibrator of aluminum (14 steps, ~30 µm of thickness by step, 99.9% Al) using a high-resolution and high-speed radiographic film (Motion Picture Fine Grain Positive Film 71337, FUJIFILM Corporation, Tokyo, Japan) according to the manufacturer’s instructions. The sample holder and the step-wedge calibrator were exposed to an X-ray generator (Softex TMR, Tokyo, Japan). The copper–nickel X-ray source (CuKa − λ = 0.154 nm) was settled at 20 kV and 20 mA for 13 min at a distance of 42 cm. A light transmitted microscope fitted with a 20× objective (Axioplan, Zeiss, Oberkochen, Germany) and a CCD camera (XC-77CE, Sony, Tokyo, Japan) attached to a computer were used for data acquisition [44]. A specific software (TMR 1.25e, Inspektor Research BV, Amsterdam, the Netherlands) was used to calculate the integrated mineral loss (∆Z %vol.µm) and the lesion depth (µm) based on the formula described by Angmar et al. [45]. The ∆Z (%vol.µm) indicates the difference between the percentage of mineral content of sound and demineralized dentin. The lesion depth is defined as the distance between the lesion surface and the dentin depth where the mineral content is ≥95% [46].

### 2.8. Cross-Sectional Hardness (CSH)

After the treatments were performed according to each group (14 groups), the dentin specimens (n = 10/group = 140) were longitudinally sectioned at the center of the surface using a double-sided diamond disk with a 22 mm diameter and a 0.10 mm thickness (7020, KG Sorensen Ind. and Com., Cotia, SP, Brazil). Each half-specimen obtained was wet-polished using silicon carbide sandpapers (#600 and #1200-grit, Wetordry™, 3M™, St. Paul, MN, USA). The specimens were kept immersed in distilled water in an incubator at 37 °C (±1 °C) using a 24-well plate culture until the analysis. The CSH measurements were performed using a microhardness testing machine (HMV-2, Shimadzu, Japan) with a Knoop indenter settled with a static load of 10 g for 10 s [35]. For each dentin specimen, two series of indentations with a distance of 100 μm between them were made at five different depths (40, 80, 120, 160 and 200 μm), totalizing ten measurements per specimen. The parameter for the initial depth measurement was the external dentin surface.

### 2.9. Fourier-Transform Raman Spectroscopy (FT-Raman)

The organic and inorganic contents of residual dentin after the treatments were measured (n = 6/group = 84). The specimens for the FT-Raman analysis were randomly assigned from the ones used for the CSH measurements. An FT-Raman microscope (Raman Scope III, Bruker Optics Inc., Karlsruhe, Germany; serial number: DE84716930) with a germanium detector and laser source was used. The dentin specimens were irradiated using an Nd: YAG laser (λ = 1064 nm) at 200 mW with a spectral resolution of 4 cm^−^^1^. To each specimen, one spectrum with a 40 μm thickness and another with a 200 μm thickness were recorded via 150 scans. Changes on the organic and inorganic contents of the dentin were analyzed by comparing the integrated areas of the Raman shifts, centered in 350–542 cm^−^^1^ (phosphate), 870–1125 cm^−^^1^ (carbonate) and 2750–3100 cm^−^^1^ (C-H bonds) [47]. The integrated area of the shifts was calculated using the LabSpec 6 Spectroscopy Suite software (Horiba Instruments Brasil Ltda., Jundiai, SP, Brazil).

### 2.10. Confocal Laser Scanning Microscopy (CLSM)

To evaluate the bacterial viability over the residual dentin after the treatments (n = 2/group = 28), CLSM images were obtained using a confocal microscope (LSM 800, Carl Zeiss, Germany; serial number: 120L-80034). To analyze the dentin surface, the specimens were immersed in 1000 μL of distilled water containing the LIVE/DEAD^®^ Baclight^TM^ Bacterial Viability Probe (Molecular Probes, Eugene, OR, USA) for 20 min. After that, the specimens were washed twice with PBS. The LIVE/DEAD^®^ dye consists of SYTO 9 (1 μL) and propidium iodide (IP; 1 μL) for each 1000 μL. SYTO 9 stains viable bacteria in green, while the PI stains non-viable bacteria in red. Excitation ranges of 488 nm (SYTO 9) and 561 nm (PI) were used.

To evaluate the bacterial viability in depth (through the dentin tubules), the dentin specimens were manually polished using silicon carbide sandpapers (#600, #1200 and #1500, Wetordry™, 3M™, St. Paul, MN, USA) until slices with a thickness of approximately 200 μm were obtained. Then, the specimens were immersed in 0.1% rhodamine B solution at 37 °C ± 1 °C for 12 h [48]. After that period, the specimens were rinsed with PBS twice and stained with LIVE/DEAD^®^ dye as previously described. Objectives of 10× magnification and excitation range of 656–700 nm for rhodamine B were used.

To evaluate the fluorescence intensity for each dye (rhodamine (blue), SYTO 9 (green) and propidium iodide (red)), the ImageJ software (Image Processing and Analysis in Java, National Institutes of Health) was used. The CLSM images were split in three to separate the fluorescence intensity emitted for each dye. After that, the whole area of the image was selected to measure the percentage of fluorescence intensity. One image was analyzed for each investigated group.

### 2.11. Statistical Analysis

The normality and homoscedasticity of the data were verified using Shapiro–Wilk and Levene tests, respectively. Two-way ANOVA (treatment and depth) followed by Tukey’s post hoc test for multiple comparisons were used for CSH and FT-Raman data analysis. All the tests were employed at a significance level of 5%. The SPSS statistical software version 22 (IBM Corporation, Nova York, NY, USA) and GraphPad Prism (GraphPad Software, San Diego, CA, USA) were used for data analysis and chart creation. The TMR data were subjected to the arithmetic mean. The CLSM images were qualitatively and quantitatively analyzed.

## 3. Results

### 3.1. Transversal Microradiography (TMR)

**Figure 1:**
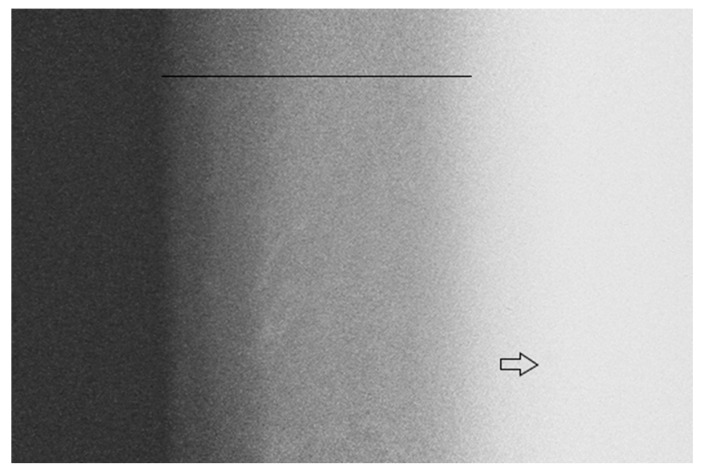
Representative TMR image of the dentin after caries-like dentin induction via the biological model. The line indicates a radiolucent zone suggesting caries-affected dentin due to the demineralization process. The arrow indicates a radiopaque zone suggesting sound dentin that was not demineralized.

Figure 1 shows a representative image of the dentin specimen after the caries-like dentin induction (7 days in a caries solution). The radiolucent area confirms that the protocol used was sufficient to induce a caries-like dentin lesion with an adequate depth and mineral loss (Table 2).

### 3.2. Cross-Sectional Hardness (CSH)

The results show that the CSH increased as a function of depth, regardless of the investigated group (*p* < 0.05). In general, decreased CSH values were observed on the first depths, followed by a gradual increase until the highest values on the final depth (200 μm) were reached.

Once the different groups were compared within the same depth, the control group (caries-like dentin lesion) showed the lowest CSH value (8.49–15.93 KHN). This result was expected since the mineral loss due to the caries-like induction dramatically decreases the CSH. Additionally, the sound dentin group showed a high CSH value, regardless of the depth (37.58–53.67 KHN). Regarding the caries removal method, the results highlight that the BUR groups (24.40–63.03 KHN) showed higher CSH values than the ULT groups (20.01–47.53 KHN), regardless of the depth or association with aPDT and bioactive glasses (*p* < 0.05).

The use of aPDT as a cavity decontamination method did not provide a significant effect on the CSH (*p* > 0.05), except for ULT and ULT + aPDT at a 200 μm depth (47.53 and 53.31 KHN, respectively; *p* < 0.05). No differences were observed between the bioactive glasses (45S5 and F18), especially when they were used in combination with carbide bur (*p* > 0.05), regardless of the depth. However, once the ultrasound was used, some differences suggest that the F18 bioactive glass had a better performance in comparison with 45S5, since higher CSH values were observed (*p* < 0.05).

Table 3 displays the mean and standard deviation of the CSH values for the different investigated groups as a function of the dentin depth.

### 3.3. FT-Raman

Three Raman shifts were calculated: phosphate, carbonate and C-H bonds. Table 4 shows the mean and standard deviation of the integrated area of the shifts for each investigated group according to the depth. For the phosphate shift, no differences among the groups and depths were observed (*p* > 0.05). For the carbonate shift, BUR + aPDT and ULT + aPDT + 45S5 were different at a 200 μm depth (0.0355 and 0.0003, respectively; *p* < 0.05). The other comparisons within the carbonate shift did not show significant differences (*p* > 0.05). Regarding the C-H bonds, no differences were found (*p* > 0.05), except between BUR and ULT + aPDT at a 40 μm depth (0.1869 and 0.0457, respectively; *p* < 0.05).

### 3.4. Confocal Laser Scanning Microscopy (CLSM)

**Figure 2:**
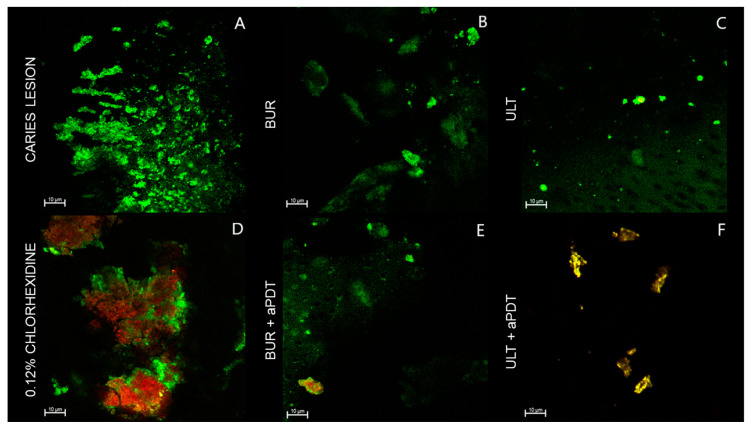
CLSM images of the remaining dentin surface after different treatments. (**A**) Negative control group: caries-like dentin lesion with no removal and no antibacterial or remineralizing treatment. (**B**) Remaining dentin after caries removal with carbide bur. (**C**) Remaining dentin after caries removal with ultrasound. (**D**) Positive control group: 0.12% chlorhexidine solution was applied over the caries-like dentin with no other treatment. (**E**) Remaining dentin after caries removal with carbide bur and decontamination with curcumin-mediated aPDT. (**F**) Remaining dentin after caries removal with ultrasound and decontamination with curcumin-mediated aPDT.

**Figure 3:**
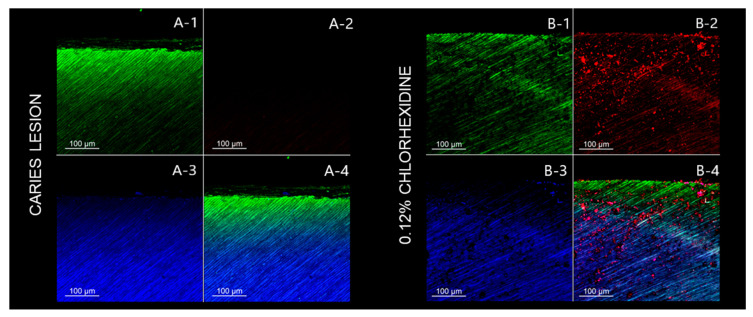
CLSM images of the remaining dentin in depth (lateral view) for control groups. (**A**) Negative control group: caries-like dentin lesion with no removal and no antibacterial or remineralizing treatment. (**B**) Positive control group: 0.12% chlorhexidine solution was applied over the caries-like dentin with no other treatment. (1) Green fluorescence indicating viable cells after staining with SYTO 9. (2) Red fluorescence indicating dead cells after staining with propidium iodide. (3) Blue fluorescence indicating the dentin after staining with 0.1% rhodamine B. (4) The image shows all three fluorescent dyes combined to highlight the penetration and viability of bacteria into the dentin. (**A-1**) Note the penetration of viable cells into the dentin. (**A-2**) Absence of dead cells, since no antibacterial treatment was performed. (**A-3**) Intertubular dentin. (**A-4**) Considerable number of viable cells penetrating into the dentin without dead cells. (**B-1**) Note the penetration of viable cells into the dentin. (**B-2**) Agglomerates of dead cells due to the antibacterial effect of chlorhexidine. (**B-3**) Intertubular dentin. (**B-4**) Although a superficial layer of viable cells is observed, several agglomerates of dead cells (red fluorescence) are observed in depth.

**Figure 4:**
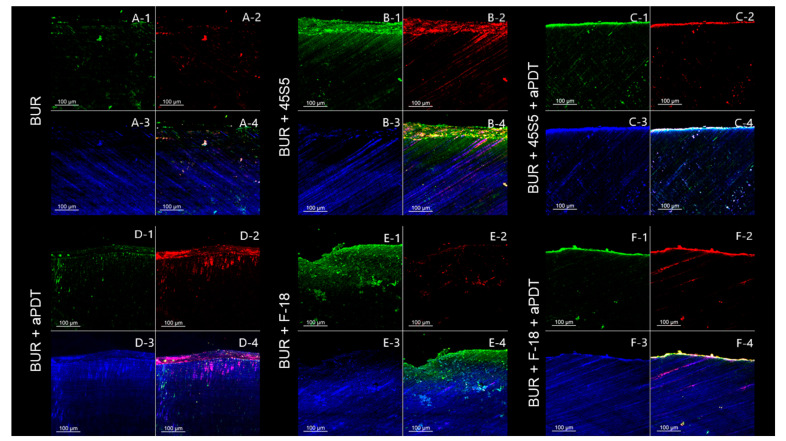
CLSM images of the remaining dentin in depth (lateral view) after different treatments using carbide bur as the removal method. (**A**) Caries removal with carbide bur. (**B**) Caries removal with carbide bur followed by the application of 45S5 bioactive glass. (**C**) Caries removal with carbide bur followed by decontamination with curcumin-mediated aPDT and application of 45S5 bioactive glass. (**D**) Caries removal with carbide bur followed by decontamination with curcumin-mediated aPDT. (**E**) Caries removal with carbide bur followed by the application of F18 bioactive glass. (**F**) Caries removal with carbide bur followed by decontamination with curcumin-mediated aPDT and application of F-18 bioactive glass. (1) Green fluorescence indicating viable cells after staining with SYTO 9. (2) Red fluorescence indicating dead cells after staining with propidium iodide. (3) Blue fluorescence indicating the dentin after staining with 0.1% rhodamine B. (4) The image shows all three fluorescent dyes combined to highlight the penetration and viability of bacteria into the dentin. (**A-1**) Few agglomerates of viable cells are observed. (**A-2**) Few agglomerates of dead cells are observed. (**A-3**) Intertubular dentin. (**A-4**) Superficial layer of viable cells is observed even after the caries removal with bur. (**B-1**,**B-2**) Disorganized layer of live and dead cells. (**B-3**) Intertubular dentin. (**B-4**) Superficial layer of viable cells with few agglomerates of dead cells. (**C-1**,**C-2**) Thin superficial layer of live and dead cells, indicating a more aggressive caries-affected dentin removal. (**C-3**) Intratubular dentin. (**C-4**) Almost no viable cells are observed. (**D-1**,**D-2**) Disorganized and superficial layer of live and dead cells. (**D-3**) Intratubular dentin. (**D-4**) Almost no viable cells are observed, especially in the most superficial area. (**E-1**,**E-2**) Disorganized layer containing viable and dead cells. (**E-3**) Intratubular dentin. (**E-4**) Viable cells are present in the most superficial area. (**F-1**,**F-2**) Fine superficial layer of live and dead cells, indicating a more aggressive caries-affected dentin removal. (**F-3**) Intratubular dentin. (**F-4**) Almost no remaining viable cells are observed.

**Figure 5:**
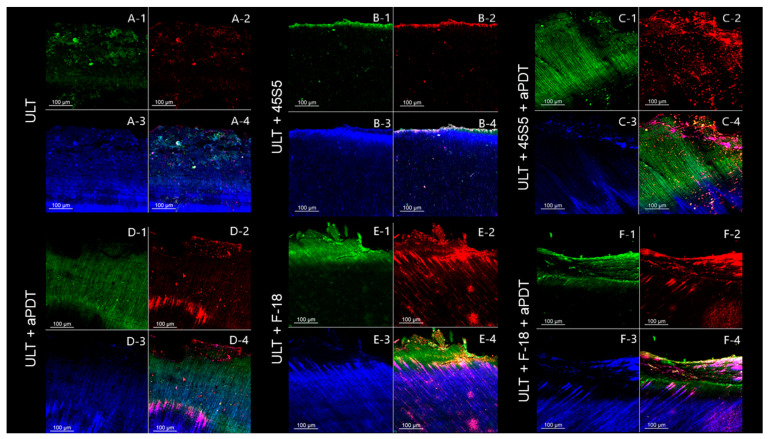
CLSM images of the remaining dentin in depth (lateral view) after different treatments using ultrasound as the removal method. (**A**) Caries removal with ultrasound. (**B**) Caries removal with ultrasound followed by the application of 45S5 bioactive glass. (**C**) Caries removal with ultrasound followed by decontamination with curcumin-mediated aPDT and application of 45S5 bioactive glass. (**D**) Caries removal with ultrasound followed by decontamination with curcumin-mediated aPDT. (**E**) Caries removal with ultrasound followed by the application of F18 bioactive glass. (**F**) Caries removal with ultrasound followed by decontamination with curcumin-mediated aPDT and application of F18 bioactive glass. (1) Green fluorescence indicating viable cells after staining with SYTO 9. (2) Red fluorescence indicating dead cells after staining with propidium iodide. (3) Blue fluorescence indicating the dentin after staining with 0.1% rhodamine B. (4) The image shows all three fluorescent dyes combined to highlight the penetration and viability of bacteria into the dentin. (**A-1**,**A-2**) Disorganized layer of viable (green) and dead (red) cells. (**A-3**) Intratubular dentin. (**A-4**) Disorganized layer due to the ultrasound vibration during the removal with almost no remaining viable cells. (**B-1**,**B-2**) Superficial layer of viable (green) and dead (red) cells. (**B-3**) Intratubular dentin. (**B-4**) Almost no remaining viable cells are observed. (**C-1**,**C-2**) Disorganized layer of viable (green) and dead (red) cells. (**C-3**) Intratubular dentin. (**C-4**) Disorganized layer containing some agglomerates of dead cells. (**D-1**,**D-2**) Disorganized layer of viable (green) and dead (red) cells. (**D-3**) Intratubular dentin. (**D-4**) Some agglomerates of dead cells are observed, mainly in the most superficial area. (**E-1**,**E-2**) Disorganized layer of viable (green) and dead (red) cells. (**E-3**) Intratubular dentin. (**E-4**) Considerable number of viable cells (green) are observed, mainly in the most superficial area. (**F-1**,**F-2**) Disorganized layer of viable (green) and dead (red) cells. (**F-3**) Intratubular dentin. (**F-4**) Considerable number of dead cells are observed, although some viable cells are also observed.

The CLSM images show the bacterial viability over the residual dentin surface after the treatments (Figure 2, Figure 3, Figure 4 and Figure 5). The caries-like dentin lesion group exhibits a predominance of green fluorescence, indicating a viable bacterial load, which was already expected since removal or antibacterial treatment were not performed (Figure 2). The positive control group (0.12% chlorhexidine) highlights the antibacterial activity of chlorhexidine, since a great red fluorescence was observed (Figure 2). The remaining dentin after removal via bur or ultrasound shows a decrease in the bacterial load, but these bacteria remain viable. Regardless of the removal method used, the aPDT application promoted bacteria inactivation, but this inactivation was more evident for the groups that used the ultrasound method (Figure 2).

The analysis throughout the dentin tubules follows the trend that was observed in the dentin surface. The negative control group (caries lesion) also shows a predominance of green fluorescence, while the positive control group (0.12% chlorhexidine) shows red fluorescence (Figure 3). In general, the BUR groups show a lower bacterial load than the ULT groups. However, the aPDT application promotes an enhanced bacteria inactivation, regardless of the removal method. The CLSM images show that the application of bioactive glasses (45S5 and F18) did not exert an antibacterial effect (Figure 4 and Figure 5).

The percentages of fluorescence intensity for each color (blue (dentin), green (live cells), and red (dead cells)) confirm the above-mentioned findings in the qualitative analysis (Table 5 and Table 6).

## 4. Discussion

This study evaluated the efficacy of different approaches (combined or isolated) for treating caries-like dentin lesions. The approaches included the use of ultrasound devices for the removal of caries lesions, aPDT for cavity decontamination and bioactive glasses for dentin remineralization. With all these experimental approaches, we attempted to provide a conservative and minimally invasive treatment.

The in vivo caries-related research is hindered by factors that include the sample number, complexity of the oral environment and bioethics issues involving human beings [46,47]. In this way, in vitro models have been used to artificially induce caries-like lesions and simulate the natural conditions as closely as possible [20,47,49,50,51,52], but without the limitations of clinical studies. Among the in vitro models, the biological model uses cariogenic bacteria and culture media, creating an ideal environment for its growth and lesion development [20,51,53]. We chose the biological model since previous studies have claimed their efficacy in reproducing artificial lesions that are comparable to natural ones [20,51,53], with a similar morphological pattern of degradation of the collagen fibrils [50]. Moreover, it was reported that the ideal time of induction is eight days [51], which is very close to that used in our study (seven days).

During the in vitro induction of a caries-like dentin, the position of the specimen is crucial to allow for active bacterial colonization over the dentin’s surface [54], simulating the formation of a real biofilm. Thus, the specimens were vertically suspended into the well, avoiding the gravity action and the formation of a sedimented cell layer [54]. In addition, two species of caries-related bacteria (*S. mutans* and *L. acidophilus*) were used to fulfill the current paradigm that the bacteria from the oral cavity are part of a complex, dynamic and interactive microbial community instead of using only one species as the etiologic agent of caries lesions [55,56]. Considering the above-mentioned information and based on the TMR results, we can state that the model and the time of induction used were enough to develop caries-like dentin lesions with an adequate depth (213.9 μm) and morphology for further experiments (Figure 1; Table 2).

All the dentin specimens included in our study underwent a surface Knoop hardness evaluation to standardize the dentin microhardness prior to the beginning of the experiments and to avoid possible bias related to the microstructure of the specimens, which could negatively interfere with the further evaluations. The surface microhardness mean value was found according to a method described in the literature [35].

An ultrasound device is an oscillatory device that operates under a frequency of above 20 kHz [11] and has been used as an alternative for cavity preparation. In our study, a prototype of an ultrasound device settled under a frequency of 27.5 kHz was developed for caries-affected dentin removal. This ultrasound device generates oscillatory movements using the piezoelectric method [57], in which the vibration results from the electric charge by the dimensional deformation of piezoelectric discs [11]. The piezoelectric effect promotes more efficiency on the energy transfer and more vibration [11], resulting in the oscillation of the diamond-coated tips, allowing for conservative cavity preparation, better removal in hard-to-reach areas, less noise, minimal thermal alteration of the tissues and lower reported pain by patients [9,10].

Curcumin was used as a photosensitizer during aPDT application because it is a low-cost and easy-to-handle natural compound that is effective in inactivating cariogenic bacteria and has also demonstrated anti-inflammatory and antioxidant effects [58,59,60]. Another advantage is related to the wavelength (400–500 nm range) that is used to irradiate this photosensitizer. This range fits to the light curing units (LCUs) employed during the photoactivation of different resin-based materials used in a dental office. Curcumin is a non-water-soluble compound, so there is a need to incorporate it into organic solvents, such as ethanol, dimethyl sulfoxide (DMSO), xylene and methanol. However, some studies have successfully investigated other options to solubilize curcumin and improve its properties such as stability and permeability [40,61,62].

The microhardness test indirectly shows a possible variation (loss or gain) on the mineral content [46]. Specifically for the CSH test, the mineral content at different depths can be obtained, providing additional information of the specimens [46]. The first null hypothesis was rejected, since our results highlight that the CSH values increased as a function of depth (Table 3). The level of structural degradation of the dentin was less aggressive as the depth increased, returning to the CSH values similar to a sound dentin on the last depths evaluated. The same CSH profile was observed in a previous study [53]. However, the CSH values in that study were lower than those obtained in our study [53]. This difference can be attributed to the caries removal methods that were used that removed the soft and infected dentin, resulting in higher CSH values.

The caries lesion process negatively affects the physical and mechanical properties of the dentin. During dentin demineralization, the hydroxyapatite is solubilized by the organic acids from the cariogenic bacteria, resulting in the dissolution of mineral crystals, a reduction in the mineral content, an increase in the porosity, and a decrease in the microhardness of the tissue [63,64]. Moreover, the literature reports that the microhardness of caries-affected dentins ranges from 25 to 40 KHN [65]. The same values were found in our study for most of the groups, highlighting the presence of caries-affected dentins even after the removal methods were performed (Table 3). However, the intergroup comparisons show that the ULT exhibited lower values than the BUR (Table 3), suggesting that the ULT provided a more conservative removal, preserving a higher amount of caries-affected dentins, which is ideal from a minimally invasive perspective.

The aPDT application after the caries removal did not show any effect on the microhardness (Table 3), which led us to state that the use of curcumin and LED irradiation did not provide alterations in the mineral content and mechanical properties of dentin. aPDT appears to be a safe and efficient antibacterial therapy that does not cause microstructural damages to the dentin. Similarly, the application of bioactive glasses (45S5 or F18) did not change the dentin’s microhardness. It seems that the ion release and the formation of hydroxyapatite were not enough to increase the CSH values. Although punctual differences suggested a better performance for F18, its clinical use still requires further investigations.

The organic and inorganic changes of dentin during the caries-like dentin lesion induction can inhibit the growth of hydroxyapatite crystals, resulting in biochemical effects. FT-Raman is a non-destructive analysis method used to evaluate the chemical composition of dentins without additional preparations of the specimens [66]. The Raman spectroscopy chemically determines different regions of the dentin via the molecular vibration of the compounds as a response of the light excitation [47,67]. In our study, no differences among the groups within the same Raman shift were observed, regardless of the depth (Table 4), which led us to accept the second null hypothesis. Ideally, we expected that the groups that received a bioactive glass application would show a larger integrated area than the others. However, the absence of a difference did not indicate the inefficacy of the bioactive glasses for promoting dentin remineralization. The FT-Raman analysis is probably not suitable for detecting the alterations due to the depth of the laser penetration [47,68]. Moreover, the autoclaving process under a high temperature (121 °C) can significantly change the organic and inorganic contents of the dentin [60], which justify the low integrated areas of the shifts.

CLSM has been used to evaluate the efficacy of antimicrobial therapies, such as aPDT [40]. The CLSM images show that curcumin-mediated aPDT inactivates the bacteria, since a greater predominance of red fluorescence was observed for the groups that received aPDT application compared to those that did not receive it (Figure 3 and Figure 4; Table 5 and Table 6). Thus, the third null hypothesis was rejected. Our results highlight aPDT as a promising and efficient cavity decontamination strategy, which can inactivate residual bacteria after caries removal and reduce their proliferation and activity. Interestingly, the caries removal using ULT followed by aPDT application showed an enhanced inactivation, suggesting a benefic effect between the combined use of ultrasound energy and aPDT. Furthermore, the CLSM images show the bacterial penetrability into the dentin tubules in a depth that is compatible with the caries lesion depth. In general, the BUR groups showed a lower bacterial load than the ULT groups, which suggests that the carbide bur removes a greater amount of caries-affected dentin as a more invasive and uncontrolled procedure. However, the aPDT application promotes enhanced bacteria inactivation, regardless of the caries removal method or bioactive glass used. Related to the bioactive glass application, neither of the bioactive glasses exerted significant bacterial inactivation, which can be explained by the short contact time over the dentin surface (5 min). It has been reported that the activity of the bioactive glasses is gradual and requires a longer time [69].

Although the artificial caries lesions are similar to natural ones, further studies using human teeth and clinical trials are crucial to better simulate in vivo conditions. Moreover, the influence of the approaches investigated in this study (ultrasound energy, aPDT and bioactive glass) on the adhesive interface using resin-based composites should also be investigated.

## 5. Conclusions

Based on the results obtained in this study, it is possible to conclude that caries-like dentin removal using ultrasound energy promotes a more conservative removal, preserving more caries-affected dentin compared to the carbide bur method. The use of curcumin-mediated aPDT as a cavity decontamination strategy inactivates the residual bacteria effectively without causing microstructural and chemical damages to the dentin. The application of bioactive glasses (45S5 and F-18) for 5 min was not enough to promote dentin remineralization.

## Figures and Tables

**Table 1:** Investigated groups.

Group	Removal Method	Cavity Decontamination Method	Dentin Remineralization Method
Caries lesion	−	−	−
Sound dentin	−	−	−
BUR	Carbide bur	−	−
BUR + aPDT	Carbide bur	Curcumin-mediated aPDT	−
BUR + 45S5	Carbide bur	−	Application of 45S5 bioactive glass for 5 min
BUR + aPDT + 45S5	Carbide bur	Curcumin-mediated aPDT	Application of 45S5 bioactive glass for 5 min
BUR + F-18	Carbide bur	−	Application of F-18 Bioglass for 5 min
BUR + aPDT + F-18	Carbide bur	Curcumin-mediated aPDT	Application of F-18 Bioglass for 5 min
ULT	Ultrasound device	−	−
ULT + aPDT	Ultrasound device	Curcumin-mediated aPDT	−
ULT + 45S5	Ultrasound device	−	Application of 45S5 bioactive glass for 5 min
ULT + aPDT + 45S5	Ultrasound device	Curcumin-mediated aPDT	Application of 45S5 bioactive glass for 5 min

**Table 2:** Mean and standard deviation values (±) of the mineral loss (vol%.µm) and caries-like dentin lesion depth (µm) after induction (7 days).

	Mineral Loss ∆Z (%vol.µm)	Depth (µm)	R (∆Z/µm)
Mean value	4929.3	213.9	23.3
Standard deviation	1049.3	49.5	3.7

**Table 3:** Mean and standard deviation values (±) of the cross-sectional Knoop microhardness (KHN) of dentin at different depths (µm) (n = 10). BUR (carbide bur), ULT (ultrasound), aPDT (antimicrobial photodynamic therapy), 45S5 (45S5 bioactive glass), F-18 (F-18 bioactive glass).

Groups	Depth (µm)				
	40	80	120	160	200
Caries lesion	8.49 ± 1.26 ^E,f^	10.97 ± 2.00 ^D,g^	11.98 ± 1,64 ^CD,h^	13.78 ± 1.15 ^BC,h^	15.93 ± 1.74 ^A,g^
Sound dentin	37.58 ± 3.96 ^D,a^	44.98 ± 1.40 ^C,a^	47.70 ± 2.73 ^B,a^	49.60 ± 2.32 ^AB,bc^	53.67 ± 4.38 ^A,cde^
BUR	24.40 ± 2.67 ^A,bc^	38.92 ± 3.50 ^B,b^	49.61 ± 6.29 ^C,ab^	57.12 ± 5.06 ^D,a^	63.03 ± 4.63 ^E,a^
BUR + aPDT	26.13 ± 2.64 ^A,b^	39.50 ± 4.49 ^B,b^	51.65 ± 3.94 ^C,a^	58.46 ± 5.27 ^D,a^	64.77 ± 6.16 ^E,a^
ULT	20.01 ± 4.00 ^A,d^	30.51 ± 7.12 ^B,cde^	35.17 ± 4.43 ^C,ef^	40.67 ± 4.28 ^D,efg^	47.53 ± 4.83 ^E,ef^
ULT + aPDT	20.38 ± 2.13 ^A,cd^	28.76 ± 3.07 ^B,de^	36.32 ± 1.87 ^C,e^	44.105 ± 2.75 ^D,de^	53.31 ± 2.47 ^E,cd^
BUR + F-18	24.69 ± 2.11 ^A,b^	32.70 ± 1.37 ^B,c^	40.73 ± 1.35 ^C,c^	49.07 ± 1.71 ^D,bc^	55.41 ± 1.79 ^E,cd^
BUR + aPDT + F-18	27.78 ± 1.95 ^A,b^	37.65 ± 1.54 ^B,b^	43.62 ± 1.50 ^C,b^	50.03 ± 1.87 ^D,b^	58.40 ± 2.11 ^E,ab^
BUR + 45S5	24.48 ± 1.63 ^A,b^	32.63 ± 1.63 ^B,cd^	39.05 ± 1.60 ^C,cd^	47.39 ± 1.00 ^D,cd^	56.61 ± 1.02 ^E,bc^
BUR + aPDT + 45S5	26.41 ± 1.27 ^A,b^	33.58 ± 1.55 ^B,c^	39.02 ± 1.15 ^C,cd^	43.26 ± 1.17 ^D,e^	52.40 ± 1.77 ^E,d^
ULT + F-18	17.34 ± 1.65 ^A,d^	23.68 ± 2.34 ^B,f^	35.1 ± 1.30 ^C,ef^	37.59 ± 1.95 ^D,g^	43.63 ± 3.88 ^E,f^
ULT + aPDT + F-18	17.75 ± 1.90 ^A,d^	25.41 ± 2.11 ^B,ef^	36.41 ± 2.16 ^C,de^	42.39 ± 1.58 ^D,ef^	49.04 ± 1.34 ^E,e^
ULT + 45S5	13.64 ± 0.92 ^A,e^	23.44 ± 2.44 ^B,f^	30.87 ± 2.11 ^C,g^	37.07 ± 1.58 ^D,g^	45.97 ± 2.95 ^E,f^
ULT + aPDT + 45S5	14.78 ± 2.17 ^A,e^	23.32 ± 2.21 ^B,f^	32.51 ± 2.13 ^C,fg^	39.49 ± 2.93 ^D,fg^	45.76 ± 3.35 ^E,f^

Different uppercase letters denote statistically significant difference among the depths within the same group (*p* ≤ 0.05). Different lowercase letters denote statistically significant difference among the groups within the same depth (*p* ≤ 0.05). Two-way ANOVA followed by Tukey post-hoc test.

**Table 4:** Mean and standard deviation (±) values of the integrated area of different Raman shifts and depths. BUR (carbide bur), ULT (ultrasound), aPDT (antimicrobial photodynamic therapy), 45S5 (45S5 bioactive glass) and F18 (F18 bioactive glass).

Groups	Raman Shifts					
	Phosphate (350–542 cm^−1^)		Carbonate (870–1125 cm^−1^)		C-H Bonds (2750–3100 cm^−1^)	
	40 µm	200 µm	40 µm	200 µm	40 µm	200 µm
Caries lesion	0.0079 ± 0.0108 ^A,a^	0.0032 ± 0.0220 ^A,a^	0.0108 ± 0.0152 ^A,a^	0.0046 ± 0.0068 ^AB,a^	0.0178 ± 0.0242 ^AB,a^	0.0035 ± 0.0338 ^A,a^
Sound dentin	0.0136 ± 0.0083 ^A,a^	0.0186 ± 0.0141 ^A,a^	0.0303 ± 0.0187 ^A,a^	0.0150 ± 0.0175 ^AB,a^	0.0389 ± 0.0160 ^AB,a^	0.0283 ± 0.0278 ^A,a^
BUR	0.0275 ± 0.0249 ^A,a^	0.0574 ± 0.0331 ^A,a^	0.2472 ± 0.1242 ^A,a^	0.2516 ± 0.1343 ^AB,a^	0.1869 ± 0.0841 ^A,a^	0.2154 ± 0.1143 ^A,a^
BUR + aPDT	0.0096 ± 0.0527 ^A,a^	0.0106 ± 0.0301 ^A,a^	0.0148 ± 0.0312 ^A,a^	0.0355 ± 0.0541 ^A,a^	0.2163 ± 0.4294 ^AB,a^	0.4988 ± 0.9863 ^A,a^
ULT	0.0373 ± 0.0254 ^A,a^	0.0128 ± 0.0088 ^A,a^	0.0284 ± 0.0282 ^A,a^	0.0480 ± 0.0350 ^AB,a^	0.0399 ± 0.0431 ^AB,a^	0.0365 ± 0.0187 ^A,a^
ULT + aPDT	0.0242 ± 0.0163 ^A,a^	0.0284 ± 0.0077 ^A,a^	0.0271 ± 0.0069 ^A,a^	0.0341 ± 0.0185 ^AB,a^	0.0457 ± 0.0064 ^B,a^	0.0660 ± 0.0971 ^A,a^
BUR + F18	0.0063 ± 0.0190 ^A,a^	0.0095 ± 0.0195 ^A,a^	0.0062 ± 0.0220 ^A,a^	0.0215 ± 0.0213 ^AB,a^	0.0102 ± 0.0416 ^AB,a^	0.0081 ± 0.0471 ^A,a^
BUR + aPDT + F18	0.0128 ± 0.0156 ^A,a^	0.0029 ± 0.0202 ^A,a^	0.0158 ± 0.0210 ^A,a^	0.0049 ± 0.0432 ^AB,a^	0.0035 ± 0.0384 ^AB,a^	0.0089 ± 0.0096 ^A,a^
BUR + 45S5	0.0097 ± 0.0199 ^A,a^	0.0046 ± 0.0148 ^A,a^	0.0066 ± 0.0257 ^A,a^	0.0004 ± 0.0303 ^AB,a^	0.0091 ± 0.0518 ^AB,a^	0.0045 ± 0.0453 ^A,a^
BUR+ aPDT + 45S5	0.0286 ± 0.0186 ^A,a^	0.0118 ± 0.0199 ^A,a^	0.0118 ± 0.0139 ^A,a^	0.0102 ± 0.0154 ^AB,a^	0.0043 ± 0.0207 ^AB,a^	0.0343 ± 0.0514 ^A,a^
ULT + F18	0.0034 ± 0.0329 ^A,a^	0.0111 ± 0.0139 ^A,a^	0.0123 ± 0.0174 ^A,a^	0.0248 ± 0.0360 ^AB,a^	0.0205 ± 0.0400 ^AB,a^	0.0196 ± 0.0278 ^A,a^
ULT + aPDT + F18	0.0036 ± 0.0173 ^A,a^	0.0030 ± 0.0260 ^A,a^	0.0147 ± 0.0339 ^A,a^	0.0008 ± 0.0327 ^AB,a^	0.0123 ± 0.0520 ^AB,a^	0.0078 ± 0.0040 ^A,a^
ULT + 45S5	0.0138 ± 0.0150 ^A,a^	0.0097 ± 0.0100 ^A,a^	0.0126 ± 0.0376 ^A,a^	0.0125 ± 0.0207 ^AB,a^	0.0022 ± 0.0694 ^AB,a^	0.0319 ± 0.0401 ^A,a^
ULT + aPDT + 45S5	0.0077 ± 0.0312 ^A,a^	0.0105 ± 0.0196 ^A,a^	0.0068 ± 0.0296 ^A,a^	0.0003 ± 0.0160 ^B,a^	0.0233 ± 0.0243 ^AB,a^	0.2790 ± 0.754 ^A,a^

Different uppercase letters denote a statistically significant difference among the groups within the same depth of the same Raman shift (*p* ≤ 0.05). Different lowercase letters denote a statistically significant difference among the depths within the same group of the same Raman shift (*p* ≤ 0.05). Two-way ANOVA followed by Tukey’s post hoc test.

**Table 5:** Fluorescence intensity measurement of CLSM images of residual dentin after different treatments.

Groups	Fluorescence Intensity (%)	
	Green (Live Cells)	Red (Dead Cells)
Caries lesion	34.62	02.50
CHX	23.26	26.05
BUR	12.92	02.21
BUR + aPDT	11.36	04.04
ULT	23.22	02.48
ULT + aPDT	02.69	04.13

**Table 6:** Fluorescence intensity (%) measurement of CLSM images of residual dentin in depth after different treatments.

Groups	Fluorescence Intensity (%)		
	Blue (Dentin)	Green (Live Cells)	Red (Dead Cells)
Caries lesion	128.89	65.10	08.06
CHX	70.83	42.97	32.68
BUR	55.60	07.92	05.56
BUR + aPDT	80.16	10.56	20.34
BUR + 45S5	57.82	34.50	24.49
BUR + F-18	86.10	30.37	04.05
BUR + aPDT + 45S5	46.57	20.19	07.37
BUR + aPDT + F-18	82.89	12.10	10.43
ULT	101.45	21.05	13.96
ULT + aPDT	62.88	42.32	28.07
ULT + 45S5	70.80	12.43	10.74
ULT + F-18	92.45	38.87	45.56
ULT + aPDT + 45S5	40.34	51.98	32.03
ULT + aPDT + F-18	69.73	20.76	31.81

## Data Availability

The data collected for this study are available from the corresponding authors upon request.
